# Jupiter’s Overturning Circulation: Breaking Waves Take the Place of Solid Boundaries

**DOI:** 10.1029/2021GL095756

**Published:** 2021-10-25

**Authors:** Andrew P. Ingersoll, Sushil Atreya, Scott J. Bolton, Shawn Brueshaber, Leigh N. Fletcher, Steven M. Levin, Cheng Li, Liming Li, Jonathan I. Lunine, Glenn S. Orton, Hunter Waite

**Affiliations:** 1 Division of Geological and Planetary Sciences, California Institute of Technology, Pasadena, CA, USA; 2 Climate and Space Sciences, University of Michigan, Ann Arbor, MI, USA; 3 Space Science, Southwest Research Institute, San Antonio, TX, USA; 4 Jet Propulsion Laboratory, California Institute of Technology, Pasadena, CA, USA; 5 School of Physics and Astronomy, University of Leicester, Leicester, UK; 6 Department of Physics, University of Houston, Houston, TX, USA; 7 Department of Astronomy, Cornell University, Ithaca, NY, USA

## Abstract

Cloud-tracked wind observations document the role of eddies in putting momentum into the zonal jets. Chemical tracers, lightning, clouds, and temperature anomalies document the rising and sinking in the belts and zones, but questions remain about what drives the flow between the belts and zones. We suggest an additional role for the eddies, which is to generate waves that propagate both up and down from the cloud layer. When the waves break they deposit momentum and thereby replace the friction forces at solid boundaries that enable overturning circulations on terrestrial planets. By depositing momentum of one sign within the cloud layer and momentum of the opposite sign above and below the clouds, the eddies maintain all components of the circulation, including the stacked, oppositely rotating cells between each belt-zone pair, and the zonal jets themselves.

## Plain Language Summary

The dark belts and bright zones that circle the planet at constant latitude, along with the jet streams on the belt-zone boundaries, are the iconic dynamical features of Jupiter’s atmosphere. But the circulation cells with rising, sinking, and cross-latitude motion are just as important because they maintain the storms and turbulent eddies. Voyager and Cassini have shown that the turbulent eddies put energy into the jet streams. We argue that the eddies also put energy into the circulation cells. They do this by generating waves that break as they propagate above and below the clouds. The breaking waves provide the essential forces that replace those that occur on planets with solid boundaries.

## 1. Introduction

Observations from Voyager and Cassini indicate that the eddy winds, which are departures from the zonal mean winds, are tending to increase the velocity difference between the eastward jets and the adjacent westward jets. This represents a transfer of momentum from one latitude to another, and it has to be balanced by a transfer in the opposite direction. Similar transfers occur in Earth’s atmosphere, but the balance is generally maintained through interaction with the solid or liquid surface below. Jupiter has no surface, so the transfer must take place entirely within the atmosphere. The possibilities include: small-scale turbulence below the resolution of the instruments, interaction of the zonal jets with the magnetic field at 1000s of km depth (Galanti et al., 2021; Kaspi et al., 2018; Schneider & Liu, 2009), and atmospheric waves that carry momentum up and down from their source regions and deposit it where they break. The third possibility is discussed here. We offer it as a hypothesis. More analysis of observations, more modeling, and more hypotheses are needed. Stimulating that effort is the main objective of this paper.

The horizontal transfer of momentum by eddies is contained in the eddy momentum flux (EMF). Let u′ and v′ be the eastward and northward residual winds after the zonal mean winds $\bar{u}(y)$ have been subtracted off, where *y* is the northward coordinate. Then the EMF is $\rho \bar{u^{\prime }v^{\prime }}$, the northward flux of eastward momentum per area per time, where *ρ* is the density and the overbar represents the zonal mean—the average with respect to longitude. The EMF was measured by tracking cloud motions in sequences of images, first by Voyager (Beebe et al., 1980; Ingersoll et al., 1981) and later by Cassini (Salyk et al., 2006). With over 200,000 velocity measurements spread over the region between ±50° of latitude, Salyk et al. (2006) obtained estimates of the mean zonal wind $\bar{u}(y)$ and the eddy wind covariance $\bar{u^{\prime }v^{\prime }}$ in latitude bands 1° wide. The winds are measured near the 1 bar pressure level (Banfield et al., 1998; Matcheva et al., 2005; Sromovsky & Fry, 2018). Although direct observations are lacking, it is likely that the EMF does not extend much deeper, because then the rate of transfer of eddy kinetic energy into energy of the zonal jets would exceed the energy supplied by solar and internal heat (Ait-Chaalal & Schneider, 2015; Liu & Schneider, 2010; Schneider & Liu, 2009).

**Figure 1.**
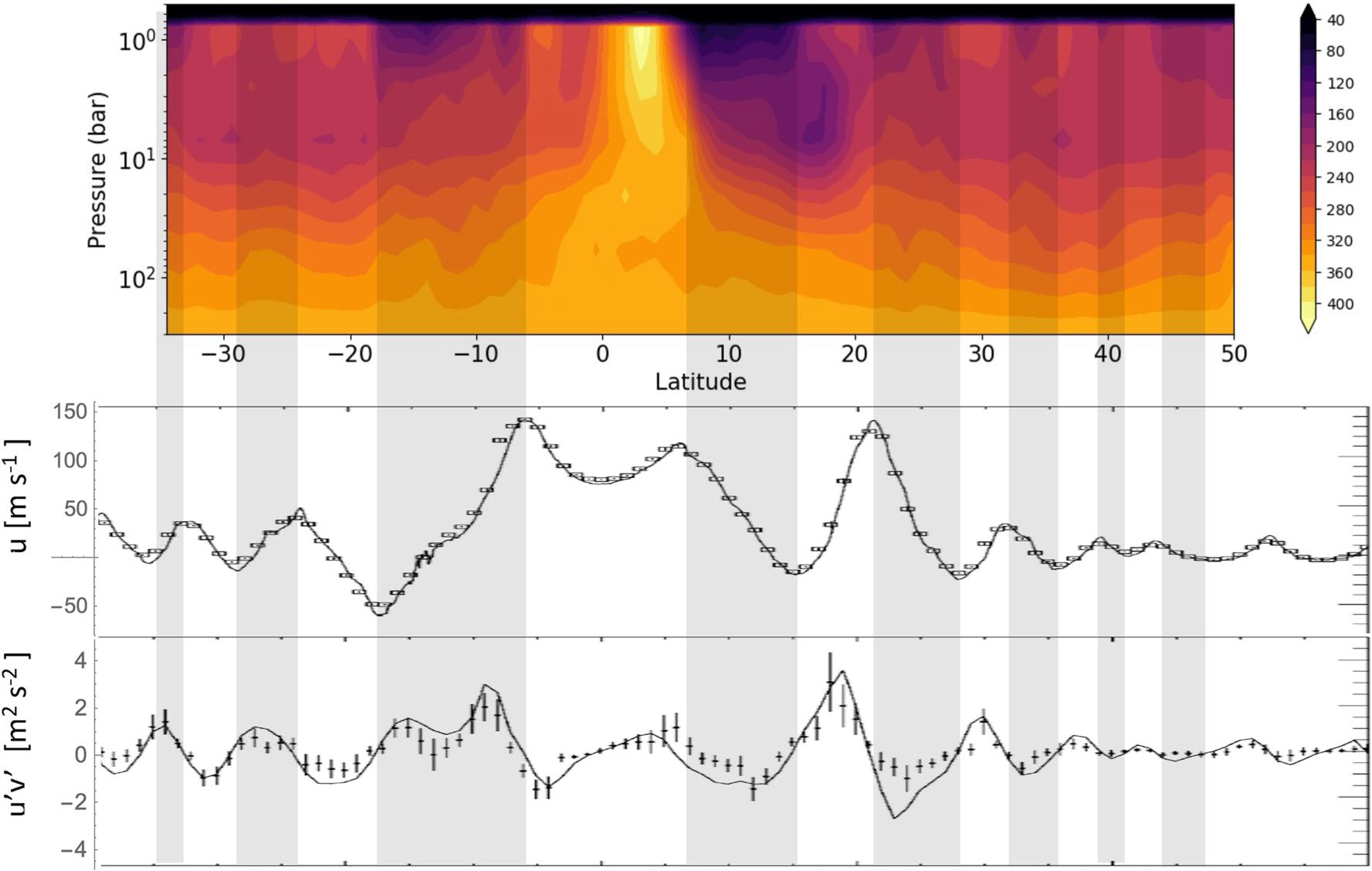
Ammonia vapor concentration (upper panel) in parts per million derived from Juno Microwave Radiometer (MWR) observations compared with dynamical features of Jupiter’s atmosphere. Belts (gray bands) and zones (white bands) are defined by the cyclonic or anticyclonic vorticity $\partial \bar{u}/\partial y$ of the zonal winds (middle panel). The eddy momentum flux (EMF, northward flux of eastward momentum) divided by the density (lower panel) is poleward in the zones and equatorward in the belts (Salyk et al., 2006). The points are $\bar{u^{\prime }v^{\prime }}$ and the smooth curve is $\partial \bar{u}/\partial y$. The MWR map differs from earlier maps (Bolton et al., 2017; Ingersoll et al., 2017; C. Li et al., 2017) because it is an average of seven north-south scans of the planet and is an inversion that uses not only the nadir brightness data but also the center-to-limb darkening data (Oyafuso et al., 2020). Notable features of the MWR map are the extreme dryness (depleted ammonia vapor) from 1 to 6 bars in the belts on either side of the equator, the ammonia increase with altitude from 1 to 6 bar both at the equator and at the zones in midlatitudes, and wavy contours implying rising and sinking motion in the belts and zones, respectively, at 40–60 bars.

Figure 1 shows $\bar{u}(y)$ in the middle panel and $\bar{u^{\prime }v^{\prime }}$ and $\partial \bar{\text{u}}/\partial \text{y}$ in the lower panel. Changes in $\bar{u}(y)$ over 20 years, from Voyager to Cassini (Limaye, 1986; Porco et al., 2003; Salyk et al., 2006) are seen at only a few latitudes. The sign of $\partial \bar{\text{u}}/\partial \text{y}$, cyclonic versus anticyclonic, defines the latitudes of the belts and zones, respectively. Figure 1 reveals that $\bar{{\text{u}}^{\prime }{\text{v}}^{\prime }}$ is positive on the south side of the eastward jets and negative on the north side, indicating that the EMF is putting eastward momentum into the eastward jets and westward momentum into the westward jets. In both cases, the effect would be to amplify the speed of the jets if there were no north-south flow to slow them down. The balance is expressed in the transformed Eulerian-mean (TEM) equations for the zonal mean eastward acceleration on page 128 of Andrews et al. (1987), hereinafter AHL:

(1a) $$\frac{\partial \bar{\text{u}}}{\partial \text{t}}-{\text{f}\bar{\text{v}}}^{*}=\rho^{-1}\nabla \cdot \text{F}$$

(1b) $$\text{F}=\left[{\text{F}}_{\text{x}},{\text{F}}_{\text{y}},{\text{F}}_{\text{z}}\right]=\left[0,-\rho \bar{{\text{v}}^{\prime }{\text{u}}^{\prime }},\rho \text{f}\bar{{\text{v}}^{\prime }\theta^{\prime }}/{\bar{\theta }}_{\text{z}}-\rho \bar{{\text{u}}^{\prime }{\text{w}}^{\prime }}\right]$$

The vector ***F*** is known as the Eliassen-Palm (EP) flux. It lies in the meridional plane and has dimensions of momentum per unit area per time. The velocity $\bar{v}*$ is the northward component of the residual mean meridional circulation. It and the vertical component ${\bar{w}}^{*}$ are derivable from a stream function. The residual circulation is a combination of that due to the eddy momentum flux and that due to the eddy heat flux. All the effects of eddy fluxes on the mean flow $\bar{u}$ are contained in the ∇ · ***F*** term. A wave that is steady, linear, frictionless, and adiabatic has ∇ · ***F*** = 0 and therefore no effect on the mean flow (Charney & Drazin, 1961). Nonzero values of ∇ · ***F*** are associated with wave generation and breaking, nonlinearity and dissipation (AHL, p. 137; Vallis, 2017).

A summary of Figure 1 is that ∂*F*_*y*_/∂*y* > 0 on the poleward sides of the zones and ∂*F*_*y*_/∂*y* < 0 on the equatorward sides. The two terms on the left of Equation 1 are the acceleration of the mean zonal wind, which is zero in steady state, and the Coriolis acceleration associated with $\bar{\text{v}}*$, where *f* = 2Ωsin*ϕ*, Ω is the planet’s rotation rate, and *ϕ* is latitude. Ignoring the vertical flux term ∂*F*_*z*_/∂*z* for the moment and recalling that *f* changes sign at the equator, balance by the Coriolis acceleration in Equation 1 implies a residual mean flow $\bar{\text{v}}*$ into each zone from the belts on either side. This flow is driven by the EMF and therefore is taking place within the clouds in a layer around the 1-bar level.

To conserve mass in the zones, there must be upwelling ${\bar{\text{w}}}^{*}>0$ above the 1-bar level and/or downwelling ${\bar{\text{w}}}^{*}<0$ below it. Upwelling in the zones above the clouds is consistent with Earth-based observations (Hess & Panofsky, 1951; Westphal, 1969) and Voyager infrared spectrometer (IRIS) observations at the 270-mbar level (Gierasch et al., 1986). The evidence includes high, thick clouds, high concentration of ammonia vapor, relatively low temperatures—a sign of upwelling in a stably stratified atmosphere, and a higher concentration of the high-temperature form of the H_2_ molecule. Downwelling in the zones below the clouds is consistent with the relative absence of lightning in the zones (Little et al., 1999). It is also consistent with the upward and downward excursions of the ammonia isolines at 40–60 bars, as seen in the top panel of Figure 1.

The horizontal flow within the clouds and the upwelling/downwelling circulation must be closed above and below the clouds by return flows from zones to belts. This led to the vertically stacked, oppositely rotating 2-cell model for each belt-zone pair (Ingersoll et al., 2000). The concept was introduced after the Galileo lightning results but without consideration of the Voyager EMF results or the Juno MWR results, although they are all qualitatively consistent. Work on the MWR results continues. Duer et al. (2021) show evidence of downwelling below the zones from 1.5 to 240 bars, with 16 belt-zone pairs from −60° to 60° latitude. Fletcher et al. (2021) documents an ammonia minimum near the 5-bar level, and Guillot et al. (2020) explains how some ammonia might escape detection at that level.

Equation 1 is capable of explaining all of the 2-cell circulation. If the EMF is confined to the clouds and the ∂F_y_/∂y part of ∇ · ***F*** is negligible above and below, then the ∂F_z_/∂z part must balance the $-\text{f}\bar{\text{v}}*$ term above and below the clouds. This could happen if a vertically propagating wave carrying momentum of the right sign were to break and deposit its momentum. In the absence of direct observations, we make a key assumption that the waves are generated within the clouds at the same levels as the EMF and have the same speed of propagation. Since the EMF eddies are accelerating both the eastward and westward jets, it is natural to assume that the phase speeds *c* of the waves are similarly bounded by the jet speeds. Most of the mesoscale waves, which have wavelengths around 300 km, are inertia-gravity waves (Orton et al., 2020), and they satisfy this criterion—their phase speeds are <50% and often <10% of the zonal flow speeds, regardless of direction (Arregi et al., 2009; Hunt & Muller, 1979; Orton et al., 2020; Simon et al., 2015). So, we choose a reference frame that has eastward jets on the poleward sides of the zones and westward jets on their equatorward sides, and we assume *c* = 0 in that frame. The goal is to see if the return flow $\bar{\text{v}}*$, driven by breaking waves above and below the clouds, is always from zones to belts.

We first consider inertia-gravity waves in Cartesian geometry with *f* = constant. Later we consider planetary waves on a beta plane. Mathematical details are in the Supporting Information S1. We assume an ideal gas and hydrostatic balance, and we use *z* = −*H*log(*p*/*p*_*s*_) as the vertical coordinate (AHL, pp. 113 and 189–192). Then the gravitational potential Φ(*x*, *y*, *z*, *t*) is a dependent variable. Minus the gradient of Φ is the acceleration due to pressure. The reference pressure p_s_, the scale height *H*, the background flow $\bar{u}$, the buoyancy frequency *N*, and the background potential temperature gradient ${\bar{\theta }}_{\text{z}}$ all are constant. The dependent variables are Φ, *θ*, and the velocity components *u*, *v*, and *w*. The perturbation quantities vary as

(2) $$\Phi (\text{x},\text{y},\text{z},\text{t})=\hat{\Phi }\exp[z/2H]\exp[ikx+ily+imz-i\text{kct}],$$

We assume large horizontal scales relative to the vertical scale, such that k^2^ ≪ m^2^, but we allow *N*^2^*k*^2^ ~ *f*^2^*m*^2^ and therefore *N*^2^ >> *f*^2^. The factor exp(*z*/2*H*) arises from the density term in the continuity equation. It ensures that the energy and momentum fluxes remain independent of height when the wave is steadily propagating. The Fourier amplitude $\hat{\Phi }$ is a function of *k*, *l*, *m*, and *c*, and the other Fourier amplitudes are proportional to it (AHL, p. 198).

In the Supporting Information S1, we show that the dispersion relation for inertia-gravity waves is

(3) $$\omega =\pm {\left(\omega_{\text{p}}^{2}+{\text{f}}^{2}\right)}^{1/2}\;\text{where}\;\omega_{\text{p}}^{2}\equiv {\text{N}}^{2}{\text{k}}^{2}{\left(\;{\text{m}}^{2}+\frac{1}{4{\text{H}}^{2}}\right)}^{-1}$$

Here, we are using $\omega =\text{k}(\text{c}-\bar{u})$. Note that *ω*^2^ > f^2^, which means that the waves are different from planetary waves and do not obey the quasi-geostrophic equations. Without loss of generality, we set *l* = 0 and we choose the plus sign in Equation 3 so that *ω* is always positive. This leaves just the two wavenumbers *k* and *m* to determine the directions of propagation, and there are four possibilities. The sign of *k* is opposite to the sign of $\left(\bar{\text{u}}-\text{c}\right)$ since $\omega =\text{k}(\text{c}-\bar{\text{u}})>0$, which means that a stationary wave, one for which *c* = 0, propagates horizontally opposite to the flow. The sign of *m* is determined by the vertical component $\hat{\text{k}}\cdot {\vec{\text{c}}}_{\text{g}}=\partial \omega /\partial \text{m}$ of the wave’s group velocity. In the Supporting Information S1, we show that upward momentum propagation, $\hat{\text{k}}\cdot {\vec{\text{c}}}_{\text{g}}>0$, is accomplished by downward phase propagation, *m* < 0, and vice versa.

**Figure 2.**
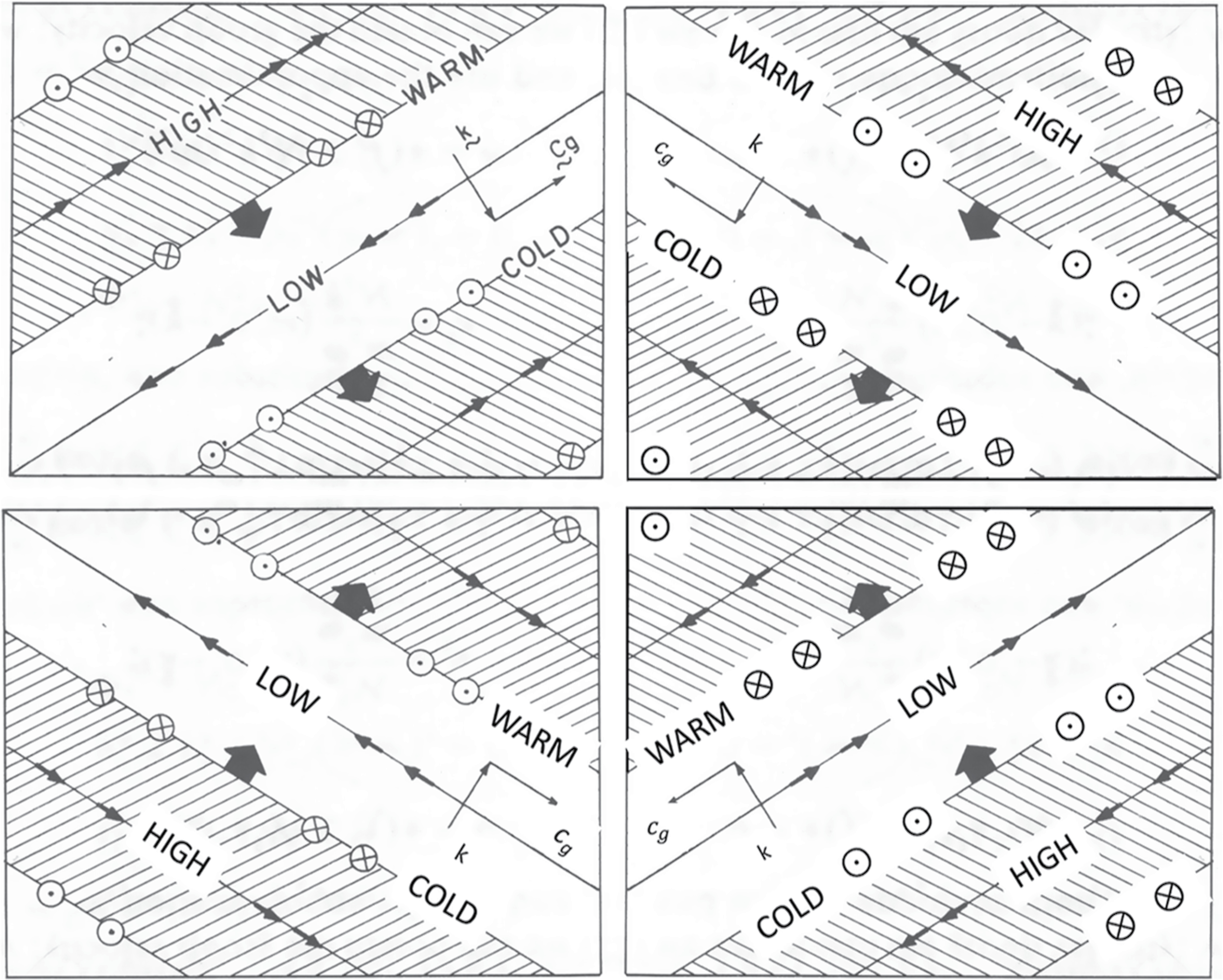
Inertia-gravity waves propagating in the *x*-*z* plane. The *x* coordinate is to the east (velocity *u*) and the *z* coordinate is vertical (velocity *w*). The left two panels show a latitude where the zonal wind $\bar{u}$ is to the west relative to the phase velocity c of the waves $\left(\bar{u}-c<0\right)$, and the right two panels show the opposite $\left(\bar{u}-c>0\right)$. The top two panels show waves that are carrying momentum upward and exerting a drag force on the flow above the source region. The bottom two panels show the opposite—a drag force below the source region. The figure shows a snapshot of each of the four wave types. The thick black arrows are in the direction of phase propagation and are perpendicular to the crests and troughs of the wave. Arrows along the crests and troughs are the fluid velocities. Phase velocity and group velocity are denoted by *k* and ***c***_*g*_, respectively. The words high and low refer to the gravitational potential at the crests and troughs. The words warm and cold refer to temperature. Circles with crosses and dots refer to poleward and equatorward flow, respectively. The figure in the upper left corner is a copy of Figure 4.19 on p. 200 of AHL. The figures in the other three corners were created by flipping and relabeling the original figure.

Figure 2 shows the four possibilities. As described earlier, we assume a stationary wave source with *c* = 0, which is an intermediate speed between those of the Jovian jets. With *c* = 0 the left two panels are for a westward jet and the right two panels are for an eastward jet. The upper two panels are for waves propagating upward from a source $\left(\hat{\text{k}}\cdot {\vec{\text{c}}}_{\text{g}}>0\right)$, with ∂*F*_*Z*_/∂*z* ∝ −*F*_*z*_, where the wave is breaking. The lower two panels are for a wave propagating downward from a source $\left(\hat{\text{k}}\cdot {\vec{\text{c}}}_{\text{g}}<0\right)$, with ∂*F*_*Z*_/∂*z* ∝ *F*_*z*_ where the wave is breaking. The general result, derived in the Supporting Information S1, is

(4) $${\text{F}}_{\text{z}}=\rho \text{f}\bar{{\text{v}}^{\prime }\theta^{\prime }}/{\bar{\theta }}_{\text{z}}-\rho \bar{{\text{u}}^{\prime }{\text{w}}^{\prime }}=\rho {\text{mkN}}^{-2}|\hat{\Phi }{|}^{2}/2$$

The ratio of ρu'w'¯ to ρfv'θ'¯/θz¯ *is ω*^2^/*f*^2^, which is greater than 1 for inertia-gravity waves, so the second term dominates in Equation 4. In other words, *F*_*z*_ has the same sign as $-\rho \bar{{\text{u}}^{\prime }{\text{w}}^{\prime }}$.

The upper left corner of Figure 2 represents a wave above the source on the equatorward side of a zone, since that is where the mean zonal wind is to the west. That corner has *k* > 0 (wave propagating to the east relative to the flow) and *m* < 0 (downward phase speed corresponding to upward group velocity). Therefore *F*_*z*_ < 0 according to Equation 4, and ∂*F*_*Z*_/∂*z* > 0 if the wave amplitude is decaying with height due to wave breaking. We call this a drag force because it provides an acceleration opposite to the zonal wind. “Form drag” is either a stress acting across wavy layers within the fluid, or else it is a stress between the fluid and wavy topography. In the former case, the divergence ∇ · ***F*** is the net zonal pressure force per unit volume. This definition holds both in oceanography (Vallis, 2017) and in meteorology (AHL, p. 137). For the upper left corner of Figure 2, it leads to $\bar{v}*$ toward the equator according to Equation 1, which is from the zone to the belt and is consistent with the return flow in the upper branch of the 2-cell circulation.

One could apply the same reasoning to the lower left corner of Figure 2, except the phase propagation is upward so that *m* > 0. As before *k* > 0, since the flow is westward. Equation 4 then gives *F*_z_ > 0 but again ∂*F*_*Z*_/∂*z* > 0, since the wave is decaying downward, and again this is a drag force on the westward wind and has $\bar{v}*$ toward the equator. This is the lower branch of the 2-cell circulation, and it is also from the zone to the belt. One can apply this reasoning to the upper right corner, corresponding to poleward side of a zone where *k* < 0 and *m* < 0, and to the bottom right corner where *k* < 0 and *m* > 0. In all four cases, standing waves (those with *c* ≈ 0) propagating upward and downward from a stationery source will balance the Coriolis force associated with the return flow. Wave breaking is always a drag force, and the return flow is always from zones to belts.

One could in principle have inertia-gravity waves whose phase velocity is faster than the zonal flow, leading to negative drag, that is, an acceleration. One prominent example is a wave moving eastward 80 m/s faster than the background zonal flow exactly at the equator and is probably a Kelvin wave (Simon et al., 2018). The equator is a special place for the dynamics of planetary atmospheres. Kelvin waves can only exist there. Perhaps the waves are generated far below the clouds where speeds are higher. Or perhaps they are a resonant response to white-noise forcing. We do not know how this wave is generated.

We now consider planetary waves on a beta plane. Again *z* = −*H*log(*p*/*p*_*s*_) is the vertical coordinate, and the gravitational potential Φ (*x*, *y*, *z*, *t*) is a dependent variable. Equation 2 still applies. We use the quasi-geostrophic equations, which are valid away from the equator when the Rossby number *Ro = U/fL* is small, where *U* and *L* are characteristic horizontal velocities and length scales, respectively. Details are in the Supporting Information S1.

The TEM equations for the zonal mean eastward acceleration, analogous to Equations 1a and 1b, are (AHL, p 129, p. 231)

(5a) $$\frac{\partial \bar{u}}{\partial t}-f{\bar{v}}^{*}=\rho^{-1}\nabla \cdot F,$$

(5b) $$F=\left[0,-\rho \bar{v^{\prime }u^{\prime }},\rho f\bar{v^{\prime }\theta^{\prime }}/\theta_{0z}\right]$$

The $-\rho \bar{u^{\prime }w^{\prime }}$ term is missing from *F*_*z*_ because |*w*′|/|*u*′| is small (of order *Ro*) compared to *H/L*. The dispersion relation for planetary waves is

(6) $$\omega =k(c-\bar{u})=-\beta k/\left[k^{2}+\left(m^{2}+1/4H^{2}\right)f^{2}/N^{2}\right].$$

This dispersion relation is different from Equation 3, which is valid for inertia-gravity waves, but remarkably, the expression for *F*_*z*_ is the same as Equation 4. For a disturbance at latitude *ϕ* on a planet rotating at rate Ω with radius *a*, one has *β* = (2Ω/*a*)cos*ϕ*, which is positive everywhere (but see below). Therefore $c-\bar{u}$ must be negative—the phase velocity must be westward relative to the flow. Figure 2 applies to planetary waves just as it applies to gravity waves when the wind is to the east relative to the phase velocity of the wave, that is, to the right side of Figure 2.

Near the centers of the westward jets there is an effective *β*, which may be negative. Two complementary criteria govern the stability of such flows, and such flows can be stable (Dowling, 1995). For planetary waves, the effective *β* is the zonal mean latitudinal gradient of potential vorticity $\bar{q}$:

(7) $$\frac{\partial \bar{q}}{\partial \text{y}}=\beta -\frac{\partial^{2}\text{u}}{\partial {\text{y}}^{2}}-\frac{1}{\rho }\frac{\partial }{\partial \text{z}}\left(\frac{\rho {\text{f}}^{2}}{\;{\text{N}}^{2}}\frac{\partial \text{u}}{\partial \text{z}}\right)$$

This expression uses the quasi-geostrophic approximation. The first two terms on the right have been measured several times, and their sum is negative at the centers of the westward jets (Beebe et al., 1980; Ingersoll et al., 1981; L. M. Li et al., 2004; Limaye, 1986; Salyk et al., 2006). Read et al. (2006) show that the third term is small in the upper troposphere, so $\partial \bar{q}/\partial \text{y}$ is still negative there. The uncertainty of the third term is greater at depth, mainly because of the uncertainty of *N*^2^ (Magalhaes et al., 2002). But if $\partial \bar{q}/\partial \text{y}$ is negative, then the left side of Figure 2 applies, and planetary waves could act as brakes on the westward jets as they do on the eastward jets.

We close with a semiquantitative test of our application of the TEM Equation 1 to Jupiter. The EMF provides a quantitative estimate of the poleward velocity. Assume the EMF dominates ∇ · ***F*** in the clouds around the 1-bar level. Treat the variation of $\bar{u^{\prime }v^{\prime }}$ from the center of the zone at 18° to the center of the belt at 24° as a half-cycle of a sinusoid, with peak amplitudes of 1 m^2^ s^−2^ in the zone and −1 m^2^ s^−2^ in the belt (Figure 1). One finds that ${\bar{v}}^{*}=-0.0033\;\text{m}\;{\text{s}}^{-1}$ on the belt-zone boundary. With that as the peak value and $\bar{v}*=0$ in the middle of the belt and the middle of the zone, it would take about 0.63 × 10^9^ s to go from ¼ of the way inside the belt to ¼ of the way inside the zone. An independent estimate of the circulation time comes from Voyager IRIS and is based on the radiative heating and cooling time needed to match the rising and sinking of parcels in the stably stratified upper troposphere. The IRIS team (Gierasch et al., 1986) found that air parcels in the zones and belts are moving upward and downward at rates of about 1.0 scale height in 10^9^ s, or 32 years. The two time scales are comparable, which is reassuring, but they do not involve mass conservation, which requires knowing the thickness (mass per unit area) of the layers where the meridional flow is taking place, and those are uncertain by factors of 2 or more.

## 2. Summary and Discussion

The eddies drive a flow from belts to zones within the clouds. We propose that the eddies balance the return flow from zones to belts by exciting waves that propagate upward and downward to levels where they are absorbed. The net zonal pressure force due to the eddies is a divergence, so Newton’s third law of motion applies. Without a solid boundary, the eddies give momentum and they take it away, but they do so at different altitudes and thereby maintain the jets and the two-tiered circulation between them. That is our hypothesis. It applies to giant planet atmospheres, which lack solid surfaces to provide a friction force. On Earth, the whole troposphere is the wave source, which is coupled to the solid and liquid planet, and the phase speed of waves propagating into the stratosphere at midlatitudes is generally slower than the mean zonal wind. Therefore, the upper right corner of Figure 2 is a broad-brush depiction of what drives the poleward stratospheric circulation at midlatitudes on Earth (AHL, chapters 5–9). We are saying that Jupiter, with its eastward and westward zonal jets and no friction with solid surfaces, could use processes depicted in all four corners of Figure 2 to drive circulation cells both above and below the clouds.

We have not discussed where the eddies get their energy. Possible sources include moist convection in the atmosphere (Gierasch et al., 2000; Ingersoll et al., 2000), baroclinic instability driven by the equator-to-pole difference in radiative heating (Liu & Schneider, 2010; Schneider & Liu, 2009), and internal heat arising from the MHD region 1000’s of km below the clouds (Cuff & Heimpel, 2018; Heimpel et al., 2016; Yadav & Bloxham, 2020). Waves propagating away from the source affect the flow where the waves are dissipating. We also have not discussed the dissipation. It could be radiation, wave instability, turbulence, or absorption at a critical layer (Andrews & McIntyre, 1976). Having *N*^2^ go to zero at depth does not necessarily cause dissipation. It could lead to evanescence (*m*^2^ < 0) and reflection of the wave, but convection provides turbulence below the clouds, and decay of the zonal winds with depth could produce critical layers. Observations of Jupiter’s gravity field indicate that the decay scale is ~3,000 km (Kaspi et al., 2018), but the observations are only sensitive to latitudes within ±25° of the equator (Galanti et al., 2021). Wave generation and dissipation are difficult subjects, even for Earth’s atmosphere. There are several types of waves and several ways of generating/dissipating them. Some waves are hard to observe directly. This paper is a first step. Next is to add numbers for the wave sources and sinks and try to account for the depth of the lower circulation cell, the value of the ammonia minimum around 6 bars, and ultimately the widths of the belts and zones and the speeds of the zonal jets.

## Supplementary Material

S.1

## Data Availability

Juno MWR data used in producing Figure 1 are available through the Planetary Atmospheres Node of the Planetary Data System (https://pds-atmospheres.nmsu.edu/cgi-bin/getdir.pl?&volume=jnomwr_1100). Analyzed data are published in Oyafuso et al. (2020).

## Key Points:

- This study proposes a dynamical mechanism that maintains the circulation cells connecting neighboring belts and zones of Jupiter
- Waves that propagate down from the cloud layer are key; when they break they produce a drag force that mimics the effect of a solid boundary
- Eddies within the clouds drive the zonal jets and probably drive the waves, thereby driving all aspects of the zonal mean circulation
