# Supplementary material for: Jupiter's Overturning Circulation: Breaking Waves Take the Place of Solid Boundaries
Source: Geophys Res Lett. 2021 Nov 28;48(23):e2021GL095756. doi: 10.1029/2021GL095756 (PMC8753638; doi:10.1029/2021GL095756)
Supplement: Supplementary file 1 — Supporting Information S1 [file GRL-48-0-s001.pdf]

AGU PUBLICATIONS

Geophysical Research Letters

Supporting Information for

Jupiter's overturning circulation: Breaking waves take the place of solid boundaries

Andrew P. Ingersoll[1], Sushil Atreya[2], Scott J. Bolton[3], Shawn Brueshaber[4], Leigh N. Fletcher[5], Steven M. Levin[4], Cheng Li[2], Liming Li[6], Jonathan I. Lunine[7], Glenn S. Orton[4], Hunter Waite[3]

[1]Division of Geological and Planetary Sciences, California Institute of Technology, Pasadena, CA 91125, USA, [2]Climate and Space Sciences, University of Michigan, Ann Arbor, MI 48109, USA, [3]Space Science, Southwest Research Institute, San Antonio, TX 78238, USA, [4]Jet Propulsion Laboratory, California Institute of Technology, Pasadena, CA 91109, USA, [5]School of Physics and Astronomy, University of Leicester, University Road, Leicester, LE1 7RH, UK, [6]Department of Physics, University of Houston, Houston, TX 77004, USA, [7]Department of Astronomy, Cornell University, Ithaca, NY 14853, USA.

Contents of this file

S1. Inertia-gravity waves

S2. Planetary waves

Introduction

This document contains derivations of the dispersion relations and the vertical component of the Eliassen-Palm flux for inertia-gravity waves and planetary waves. The results of the derivations are in the main text, and there is some overlap with this document. The derivations are similar to those in Andrews Holton and Leovy (1987), hereinafter AHL, but the geometry considered here−upward and downward propagation, eastward and westward phase speeds relative to the flow−are more general. AHL consider pure internal gravity waves without rotation at the same level of detail as that here, but the detail for inertia-gravity waves is greater here than in AHL.

S1. Inertia-gravity waves

We consider inertia-gravity waves in Cartesian geometry with f = constant. We assume an ideal gas and hydrostatic balance, and we use z = − H log(p/p_s) as the vertical coordinate (AHL p. 189-192). Then the gravitational potential Φ(x, y, z, t) becomes a dependent variable. Minus the gradient of Φ is the acceleration due to pressure. The basic state density is $\rho = \rho_s \exp[-z/H]$. The scale height H, the reference pressure p_s and the reference density ρ_s are prescribed constants. The background flow $\bar{u}$ is a constant, independent of the coordinates and time. The buoyancy frequency N and the background potential temperature gradient $\bar{\theta}_z$ are assumed to be constant as well. We use subscripts for derivatives. For small amplitude disturbances the equations are (AHL p. 198)

$$u'_t + \bar{u}u'_x - fv' + \Phi'_x = 0, \quad \text{(S1a)}$$

$$v'_t + \bar{u}v'_x + fu' + \Phi'_y = 0, \quad \text{(S1b)}$$

$$\theta'_t + \bar{u}\theta'_x + w'\bar{\theta}_z = 0 \quad \text{(S1c)}$$

$$N^2\theta'/\bar{\theta}_z - \Phi'_z = 0. \quad \text{(S1d)}$$

$$u'_x + v'_y + \rho^{-1}(\rho w')_z = 0, \quad \text{(S1e)}$$

Equations (S1a) and (S1b) are the horizontal momentum equations, (S1c) is the heat equation, (S1d) is hydrostatic balance, and (S1e) is the continuity equation. Φ varies as

$$\Phi(x, y, z, t) = \widehat{\Phi} \exp[z/2H] \exp[ikx + ily + imz - ikct], \quad \text{(S2)}$$

where c is the phase velocity of the wave in the x-direction. Consistent with hydrostatic balance we are assuming large horizontal scales relative to the vertical scale, such that $k^2 \ll m^2$ and $N^2 >> f^2$, but we allow $N^2k^2 \sim f^2m^2$.The factor exp(z/2H) arises from the density term in the continuity equation. That factor ensures that the energy and momentum fluxes remain independent of height when the wave is steadily propagating. The Fourier amplitude $\widehat{\Phi}$ is a function of k, l, m and c, and the other Fourier amplitudes are proportional to it (AHL p. 198):

$$\hat{u} = (\omega^2 - f^2)^{-1}(\omega k + ilf)\widehat{\Phi} \quad \text{(S3a)}$$

$$\hat{v} = (\omega^2 - f^2)^{-1}(\omega l - ikf)\widehat{\Phi} \quad \text{(S3b)}$$

$$\hat{\theta}/\bar{\theta}_z = N^{-2}\left(im + \frac{1}{2H}\right)\widehat{\Phi} \quad \text{(S3c)}$$

$$\hat{w} = -\frac{\omega}{N^2}\left(m - \frac{i}{2H}\right)\widehat{\Phi} \quad \text{(S3d)}$$

Here we are using $\omega = k(c - \bar{u})$, which obeys the dispersion relation.

$$\omega = \pm\left(\omega_p^2 + f^2\right)^{1/2} \quad \text{where} \quad \omega_p^2 \equiv N^2k^2\left(m^2 + \frac{1}{4H^2}\right)^{-1} \qquad \text{(S4)}$$

Without loss of generality we set $l = 0$ and we choose the plus sign in (S4) so that ω is always positive. This leaves just the two wavenumbers, k and m, to completely determine the wave properties, at least in a qualitative sense. The sign of k is opposite to the sign of $(\bar{u} - c)$, since $\omega = k(c - \bar{u})$ is positive, Note that $\omega^2 > f^2$, which means that the period of the waves at mid-latitude relative to the flow is shorter than the planet's rotation period. Longer periods are possible close to the equator. The sign of m is determined by the vertical component $\hat{k} \cdot \vec{c}_g$ of the group velocity

$$\hat{k} \cdot \vec{c}_g = \frac{\partial\omega}{\partial m} = -\mathrm{m}\left(m^2 + \frac{1}{4H^2}\right)^{-1} \omega_p^2\left(\omega_p^2 + f^2\right)^{-1/2} \qquad \text{(S5)}$$

A wave carrying momentum upward must have $\partial\omega/\partial m > 0$, so it must have $m < 0$ corresponding to downward phase propagation. Similarly, a wave carrying momentum downward must have $\partial\omega/\partial m < 0$ and $m > 0$ corresponding to upward phase propagation. Figure 2 shows the four possibilities. On the left half, k is positive and the wave is propagating to the east relative to the flow. On the right half, k is negative. On the top half, m is negative and the phase propagation is downward. On the bottom half, m is positive and the phase propagation is upward.

The TEM equation for inertia gravity waves is (AHL p. 128)

$$\frac{\partial \bar{\mathrm{u}}}{\partial \mathrm{t}} - \mathrm{f}\bar{v}^* = \rho^{-1}\nabla \cdot \mathbf{F} \qquad \text{(S6a)}$$

$$\mathbf{F} = [F_x, F_y, F_z] = [0,\ -\rho\overline{\mathrm{v'u'}},\ \rho\mathrm{f}\overline{\mathrm{v'\theta'}}/\bar{\theta}_\mathrm{z} - \rho\overline{u'w'}] \qquad \text{(S6b)}$$

If f were zero, the acceleration of the mean zonal flow would be entirely due to the $\rho\overline{u'w'}$ term in (S6b). But with $f \neq 0$, the wave includes an eddy wind v' that varies in phase with the potential temperature fluctuation θ' to produce a mean eddy heat flux proportional to $\overline{v'\theta'}$. Its horizontal divergence produces a mean vertical velocity in the heat equation, which, by mass conservation, produces an additional mean acceleration in the zonal momentum equation.

To evaluate the terms, one must first take the real parts of the complex exponentials in Equations (S3), reducing them to sines and cosines, and then average over a full cycle of the wave.

$$\rho f\overline{v'\theta'}/\bar{\theta}_z = -\rho(\omega^2 - f^2)^{-1}N^{-2}f^2mk\left|\hat{\Phi}\right|^2/2 \qquad \text{(S7a)}$$

$$-\rho\overline{u'w'} = \rho(\omega^2 - f^2)^{-1}N^{-2}\omega^2mk\left|\hat{\Phi}\right|^2/2 \qquad \text{(S7b)}$$

$$F_z = \rho f\overline{v'\theta'}/\bar{\theta}_z - \rho\overline{u'w'} \ = \rho mkN^{-2}\left|\hat{\Phi}\right|^2/2 \tag{S7c}$$

Equation (S7c) is remarkable, first because the Coriolis parameter does not appear in the expression for F_z and second because the same expression governs pure internal gravity waves (AHL p. 191), inertia-gravity waves, and planetary waves on a beta-plane (AHL p. 231), as we will show in the next section. Moreover, Equation (S7c) implies a drag force for all four cases in Figure 2. These four cases are discussed in the main text and in the caption to Figure 2.

S2. Planetary waves

For planetary waves, the equations analogous to (S1a,bc) are (AHL p. 119)

$$-\mathrm{f}v' = -\Phi'_{\mathrm{x}} \qquad \mathrm{f}u' = -\Phi'_{\mathrm{y}} \qquad \theta'/\theta_{0\mathrm{z}} = \Phi'_{\mathrm{z}}/\mathrm{N}^2) \tag{S8a,b,c}$$

These are the equations for geostrophic balance, which are valid away from the equator when the Rossby number *Ro = U/fL* is small, where *U* and *L* are characteristic horizontal velocities and length scales, respectively. The time evolution of the flow is determined by the quasi-geostrophic vorticity equation (AHL p. 122)

$$q'_t + \bar{u}q'_x + \beta\psi'_x = 0 \qquad \text{where } \psi' = \Phi'/f \tag{S9a,b}$$

$$q' = \psi'_{xx} + f\rho^{-1}(\rho\theta'/\bar{\theta}_z) = \psi'_{xx} + \rho^{-1}(\rho f^2/N^2\psi'_z)_z \tag{S9c}$$

Here ψ' is the streamfunction associated with Equations (S9a,b). A streamfunction is justified if the flow is horizontally incompressible, which follows when *Ro* << 1 and *L* << *a*, where *a* is the radius of the planet. As before, we are assuming zero for the north-south wavenumber *l*. The square of the buoyancy frequency N^2 is computed from the reference temperature profile $\bar{T}$(z) and the corresponding potential temperature gradient $\bar{\theta}_z$(z). See AHL Equations (3.2.12) and (3.2.13).

The dispersion relation for planetary waves is different from that for gravity waves

$$\omega = k(c - \bar{u}) = \frac{-\beta k}{[k^2 + (m^2 + 1/4H^2)f^2/N^2]} \tag{S10}$$

$$\frac{\partial\omega}{\partial m} = \frac{2\beta m k f^2/N^2}{[k^2 + (m^2 + 1/4H^2)f^2/N^2]^2} \tag{S11 }$$

The TEM equations for the zonal mean eastward acceleration for geostrophic flow, analogous to Equations (S6a,b), are (AHL p 129, p. 231)

$$\frac{\partial \bar{u}}{\partial t} - f\bar{v}* = \rho^{-1}\nabla \cdot \mathbf{F} \quad \text{(S12a)}$$

$$\mathbf{F} = [0,\ -\rho\overline{v'u'},\ \rho f\overline{v'\theta'}/\theta_{0z}] \quad \text{(S12b)}$$

The $-\rho\overline{u'w'}$ term is missing from F_z because $|w'|/|u'|$ is small (of order *Ro*) compared to *H/L* . That term is not small for inertia-gravity waves. Nevertheless, using complex exponentials as in Equation (S3) and averaging over a cycle one obtains the same expression for F_z as (S7c) (AHL p. 128).

$$F_z = \rho mkN^{-2}|\hat{\Phi}|^2/2 \quad \text{(S13)}$$
